# Antiviral Protein of *Momordica charantia* L. Inhibits Different Subtypes of Influenza A

**DOI:** 10.1155/2013/729081

**Published:** 2013-07-09

**Authors:** Viroj Pongthanapisith, Kazuyoshi Ikuta, Pilaipan Puthavathana, Wichet Leelamanit

**Affiliations:** ^1^Department of Biochemistry, Faculty of Pharmacy, Mahidol University, 447 Sri Ayudthya, Rajathevi, Bangkok 10400, Thailand; ^2^Department of Virology, Research Institute for Microbial Diseases, Osaka University, 3-1 Yamadaoka, Suita, Osaka 565-0871, Japan; ^3^Faculty of Medicine Siriraj Hospital, Mahidol University, 2 Prannok Road, Bangkoknoi, Bangkok 10700, Thailand

## Abstract

The new antiviral activity of the protein extracted from *Momordica charantia* was determined with different subtypes of influenza A. The protein was purified from the seed of *M. charantia* using an anion exchanger and a Fast Protein Liquid Chromatography (FPLC) system. At the concentration of 1.401 mg/mL, the protein did not exhibit cytotoxicity in Madin-Darby canine kidney cells (MDCK) but inhibited 1 × 10^5^ FFU influenza A/PR/8/34 H1N1 virus at 56.50%, 65.72%, and 100% inhibition by the protein treated before the virus (pretreated), the protein treated alongside with the virus (simultaneously treated), and the protein treated after the virus (posttreated) during incubation, respectively. Using 5, 25, and 100 TCID_50_ of influenza A/New Caledonia/20/99 H1N1, A/Fujian/411/01 H3N2 and A/Thailand/1(KAN-1)/2004 H5N1, the IC_50_ was calculated to be 100, 150, and 200; 75, 175, and 300; and 40, 75, and 200 **μ**g/mL, respectively. Our present finding indicated that the plant protein inhibited not only H1N1 and H3N2 but also H5N1 subtype. As a result of the broad spectrum of its antiviral activity, this edible plant can be developed as an effective therapeutic agent against various and even new emerging subtypes of influenza A.

## 1. Introduction

Influenza A viruses have continued to be a significant public health concern with epidemics responsible for serious morbidity and mortality. Epidemics of influenza occur almost every year according to an antigenic drift of the two viral surface glycoproteins, hemagglutinin (HA), and neuraminidase (NA). Currently, sixteen hemagglutinin subtypes (H1–H16) and nine neuraminidase subtypes (N1–N9) have been recognized [1]. In the year 1918, the highly pathogenic strains of influenza A virus emerged in an unpredictable manner, causing the death of 20–40 million people worldwide [2, 3]. Pandemic strains of the virus usually possess antigenically different, novel glycoproteins, causing the limitation of duration and cross strain protection of currently available vaccines. For example, the emerging strains of avian influenza viruses (H5N1) appeared in humans in Hong Kong in 1997 [4]. These viruses had an extremely high virulence in humans, killing 6 out of 18 infected individuals. Recently, the outbreak of influenza A/H5N1 infection has occurred among poultry in Asia and had transmission to 499 people in 15 countries with a high case fatality rate of approximately 60% [5]. According to the report of WHO on April 14, 2013, a new influenza virus H7N9 emerges in eastern China and possibly spreads into Vietnam, causing at least 13 deaths among 60 infections.

Presently, the two inhibitors of the M2 protein, amantadine, and rimantadine and the two neuraminidase inhibitors, zanamivir and oseltamivir are the available anti-influenza A agents [6]. However, up to 30% of individuals who received amantadine or rimantadine excreted viral resistant strains [7, 8]. Additionally, many reports indicated that neuraminidase inhibitor resistant strains arose rapidly in the presence of therapeutic agents [9–13]. Consequently, much attention has been focused for the development of new antiviral drugs for the effective treatment and overcoming the resistant viruses. Many researchers are attempting to find new anti-influenza agents both from chemically synthesized and naturally active compounds [14–17]. Among the medicinal plants investigated, *Momordica charantia* L. that has been reported to contain many potent antiviral activities that might be a good candidate. For instance, the proteins from this plant strongly inhibit several viruses including hepatitis B virus, dengue virus, herpes simplex virus, and Human Immunodeficiency Virus [18–21]. *M. charantia*, an edible vegetable belonging to the Cucurbitaceae family, is commonly cultivated in Africa and Asia and is known as a bitter melon or bitter ground. It has been traditionally used as folk medicines for several ailments such as antidiabetic, antitumor, anthelmintic, antimalarial, and antiviral agents [22]. For this current work, we have determined the new antiviral activity of the protein of *M. charantia* with various influenza A subtypes including influenza A/New Caledonia/20/99 H1N1, A/Fujian/411/01 H3N2, and A/Thailand/1 (KAN-1)/2004 H5N1. Our discovery has clearly indicated that the protein of *M. charantia* has an effective, broad antiviral activity against different subtypes of influenza A.

## 2. Materials and Methods

### 2.1. Plant Materials

The ripe fruits of *M. charantia* were purchased from a local market in Bangkok during January-February 2006. Briefly, the seeds were cleaned and dried by removing red tissue covers. Then, they were added with 10 mM sodium phosphate buffer pH 6.3 (PBS) containing 0.15 M NaCl with a ratio of 2 mL buffer for a gram of seeds and blended with a homogenizer for 5 minutes to form fine emulsion at 4°C. The emulsion was stirred at 4°C for 1 hour, filtrated through double cheesecloth, and centrifuged at 12,000 ×g for 10 minutes at 4°C. The supernatant was called the crude protein fraction.

### 2.2. Purification of the Protein by Fast Protein Liquid Chromatography

The crude protein was purified using a Hitrap Q anion exchanger equipped with a Fast Protein Liquid Chromatography (FPLC) system (Amersham Pharmacia Biotech, USA). Briefly, the crude protein fraction was filtrated with a syringe filter (0.45 *μ*m) and applied to the FPLC at 4–10°C in a cool cabinet. The column was washed with PBS containing 1 N NaCl, and the protein was eluted by varying the pH and ionic strength. The proteins migrated under an electric field with the pH gradient corresponding to their pI. The UV detector was set at 280 nm and the fraction size of the proteins was collected for each 1 mL. The desirable peak of the protein fraction recorded by the UV detector was collected, and the protein sample was divided into small 0.5 mL tubes and kept at 4°C for further investigation.

### 2.3. Protein Concentration Determination and SDS-PAGE Analysis

The protein concentration was determined by Bradford assay with Coomassie Plus Protein Assay Reagent (PIERCE, USA) using a spectrophotometer (Spectronic 3000 array spectrophotometer, USA). SDS-PAGE was performed according to the method described by Laemmli using the Bio-Rad Mini Protein apparatus (Bio-Rad, USA). Normally, 15% separating and 5% stacking gel with 100 constant voltage were used.

### 2.4. Cells and Viruses

MDCK cells was obtained from Virology Department, Microbial Research Institution, Osaka University (Riken cell bank), and maintained in minimal essential medium (MEM) supplemented with 10% fetal bovine serum, 1% sodium pyruvate, 1% MEM nonessential amino acids, 1% penicillin-streptomycin solution, and 1% glutamine (Invitrogen). The influenza virus A/PR/8/34 H1N1 (PR 8) was also from Virology Department, Research Institution Microbial Disease, Osaka University. Influenza A/New Caledonia/20/99 H1N1, A/Fujian/411/01 H3N2, and A/Thailand/1(KAN-1)/2004 H5N1 subtypes were provided from Virology Department, Siriraj Medicine Faculty, Mahidol University.

### 2.5. Cytotoxicity of Proteins by Cell Proliferation Assay

The cell proliferation assay kit (CHEMICON International) was used for fast and sensitivity quantification of cell proliferation and viability. The assay was based on the cleavage of tetrazolium salt WST-1 to formazan by cellular mitochondrial hydrogenases. One hundred *μ*L (1 × 10^5^ cells) of MDCK cells were added in each well of a 96-well plate. After incubation at 37°C, 5% CO_2_ for 24 hours, twofold serial dilutions of the protein were added and incubated at 37°C, 5% CO_2_ for 24 hours. Ten microliters of WST-1/ECS solution were added in each well and then incubated at 37°C, 5% CO_2_ for 1 hour. The number of viable cells was measured using a colorimetric assay system that measured the reduction of tetrazolium salt to formazan at an absorbance of 450 nm.

### 2.6. Detection of Influenza Virus A/PR/8/34 (H1N1) (PR8) Infection in MDCK Cells by an Immunofluorescence Assay

The infection of influenza virus A/PR/8/34 in MDCH cells was determined by an immunofluorescence assay. A 24-well plate was added with 1000 *μ*L/well (1 × 10^5^ cells) of MDCK cells and incubated at 37°C for 24 hr. The cells were then added with 0.175 to 1.401 mg/mL of the protein sample and incubated at 37°C for 1 hour. The plate was washed with PBS buffer twice. Then, 1 × 10^5^ focus-forming units/mL (FFU/mL) of the influenza virus A/PR/8/34 was inoculated in the wells, and the plates were further incubated at 37°C for 1 hr. After incubation, the cells were fixed with 500 *μ*L of 4% paraformaldehyde. The infected cells were identified with the nucleoprotein antibody (150 *μ*L/well) of influenza A virus (anti-NP : PBS = 1 : 1000) and incubated at room temperature for 30 min. Finally, 150 *μ*L of fluorescein isothiocyanate conjugated goat anti-mouse (anti-FITC : PBS = 1 : 500) were added in each well. Influenza A-infected cells were observed under a fluorescence microscope.

### 2.7. Detection of Influenza Virus A/PR/8/34 Infection in MDCK Cells by a Peroxidase Antiperoxidase (PAP) Staining Method

After inoculation with influenza virus A/PR/8/34, MDCK cells were incubated at 37°C for 24 hours. The infected cells were fixed with methanol for 10 minutes and dried at room temperature. Fifty *μ*L of the first antibody (Mouse IgG to influenza NP : PBS = 1 : 1000) was added in each well, and the plates were incubated at 37°C for 30 minutes. After incubation, the plates were washed with PBS buffer twice. Fifty *μ*L of the second antibody (Rabbit IgG to Mouse IgG : PBS = 1 : 1000) was added in each well, and the plates were incubated at 37°C for 30 minutes. The plates were washed twice with PBS buffer. Finally, 50 *μ*L of the third antibody (Goat IgG to rabbit IgG : PBS = 1 : 500) was added in each well, and the plates were incubated at 37°C for 30 minutes and washed twice with PBS buffer. After washing, 50 *μ*L of PAP solution (diluted with PBS to 1 : 1000) was added in each well, and the plates were incubated at 37°C for 30 minutes. Simultaneously, DAB substrate solution was prepared by adding approximately 2 mg of DAB and 2 *μ*L of H_2_O_2_ in 10 mL of PBS. After washing, 50 *μ*L DAB substrate solution was added in each well. The plates were incubated at room temperature for 20 minutes until the solution was changed to an umber color. After the plates were washed with tap water and dried at room temperature, the number of infected cells (foci) was counted under a light microscope.

### 2.8. Determination of the Incubation Time Effect of the Protein on Influenza Virus A/PR/8/34

In each experiment, 1 × 10^5^ MDCK cells were plated in each well of a 96-well plate. For the pretreated assay, the cells were added with twofold serial dilutions of different protein (called the pretreated protein) concentrations ranging from 0.17 to 1.40 mg/mL and further incubated at 37°C 5% CO_2_ for 1 hour. After washing with PBS twice, the cells were inoculated with 1 × 10^5^ FFU/mL (1 MOI) of influenza virus A/PR/8/34 and incubated at 37°C 5% CO_2_ for 24 hours. After incubation, the number of infected cells was determined by PAP staining assay as mentioned above.

For the simultaneously treated assay, the protein (called the simultaneously treated protein) was added immediately after the cells were inoculated with the virus. For the posttreated assay, the protein (called the posttreated protein) was added after the cells were preinoculated with the virus for one hour. The following steps were the same as described for the pretreated assay.

### 2.9. Determination of IC_50_ of the Protein on Influenza A/New Caledonia/20/99 H1N1, A/Fujian/411/01 H3N2, and A/Thailand/1(KAN-1)/2004 H5N1

The IC_50_ values of the protein on different subtypes of influenza A were determined using 100, 25, and 5 tissue culture infectious dose_50_ (TCID_50_) of influenza A/New Caledonia/20/99 H1N1, A/Fujian/411/01 H3N2, and A/Thailand/1(KAN-1)/2004 H5N1. Briefly, various protein concentrations ranging from 12.5 to 400 *μ*g/mL were added to each well containing 1 × 10^5^ cells of MDCK cells. After incubation at 37°C, 5% CO_2_ for 1 hour, the cells were individually inoculated with 1 × 10^5^ FFU/mL of different subtypes of influenza A and further incubation overnight. The infected cells were individually identified with nucleoprotein antibody against different influenza A viruses using an immunofluorescence assay as mentioned previously. The IC_50_ was determined by graphically plotting the inhibition of viral growth as a function of the protein concentrations.

### 2.10. Statistical Analysis

Student's *t*-tests or one-way ANOVA were used for statistical analysis. In all cases, *P* values <0.05 were considered statistically significant.

## 3. Results

### 3.1. Purification and Characterization of the Protein from *M. charantia*

**Figure 1:**
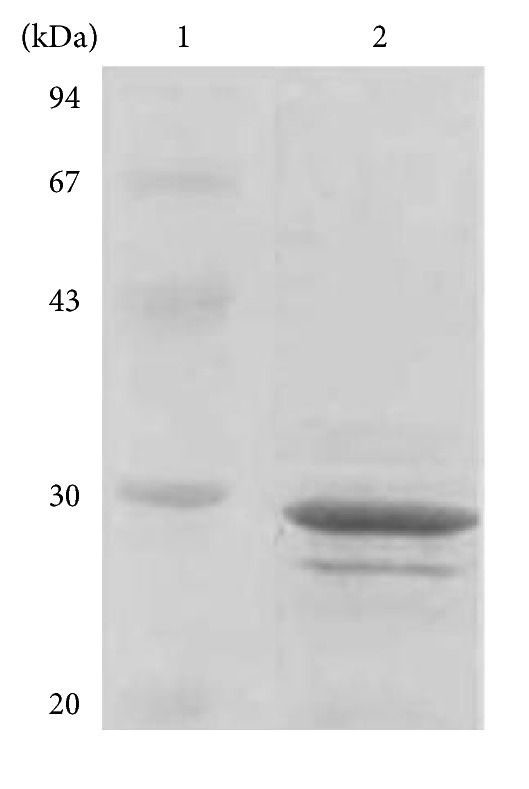
SDS-PAGE of the proteins isolated from *M. charantia*. The proteins were analyzed in 12% SDS-PAGE. Lanes 1 and 2 are molecular weight markers and the protein fraction after the FPLC separation, respectively.

After being purified with a Hitrap Q anion exchanger and a Fast Protein Liquid Chromatography (FPLC) system, the majority of the protein band appeared on the SDS-PAGE was of 30 kDa (Figure 1). This partially purified protein was used to determine its biological activities.

### 3.2. Cytotoxicity of the Protein from *M. charantia*

**Figure 2:**
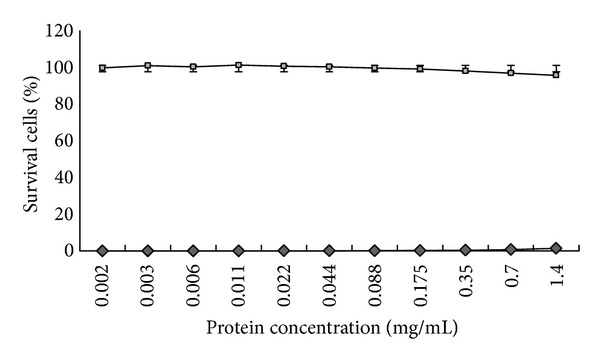
The cytotoxicity test of the protein of *M. charantia* on MDCK cells. The twofold serial dilution of the protein concentration ranging from 0.002 to 1.40 mg/mL was used in the experiment.

To evaluate the cytotoxicity effect of extracted proteins, MDCK cells were grown in different dilutions of the protein for 24 hours. The protein concentration ranging from 0.002 to 1.40 mg/mL did not exhibit any cytotoxicity in MDCK cells (Figure 2).

### 3.3. The Protein Effectively Inhibited Influenza A/PR/8/34 H1N1 Demonstrated in MDCK Cells

**Figure 3:**
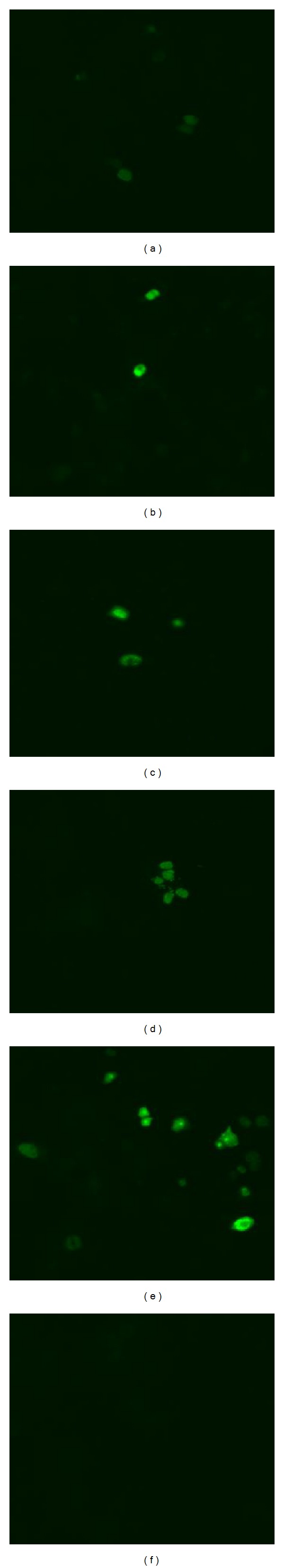
Determination of the inhibitory effect of the protein of *M. charantia* against influenza virus A/PR/8/34 (H1N1) infected in MDCK cells. The twofold dilution of the pretreated protein of 1.4 to 0.175 mg/mL ((a)–(d), resp.) was used on the assay. The viral infected cell (e) and noninfected cell (f) were used as the positive and negative controls, respectively.

The twofold serial dilutions of the extracted protein ranging from 0.0175 to 1.40 mg/mL were applied to determine the inhibitory effect against influenza virus A/PR/8/34 H1N1 at 0.1 MOI in 1 × 10^5^ MDCK cells (Figure 3). The protein (the pretreated protein) was added in each well for one hour before adding the virus. By using the immunofluorescence assay, the viral infection in MDCK was clearly reduced, especially at the protein concentration of 1.40 mg/mL (Figure 3(a)).

### 3.4. Time Incubation Effect of the Protein on Influenza Virus A/PR/8/34

**Figure 4:**
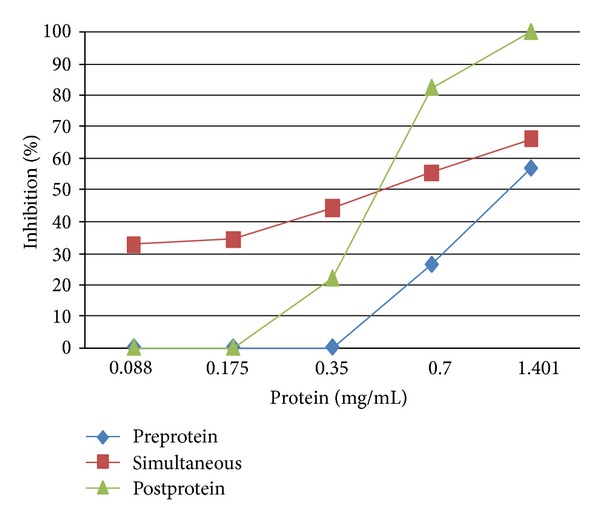
Determination of time incubation effect of the protein on influenza A/PR/8/34. The twofold serial dilutions of 0.088 to 1.40 mg/mL of the pretreated, simultaneously treated, and posttreated proteins of were individually added in the viral infected cells.

In order to determine the time incubation effect of the protein on influenza virus A/PR/8/34, the twofold serial dilution of 0.088 to 1.40 mg/mL of the pretreated, simultaneously treated, and posttreated proteins were individually added in the viral infected cells. The results indicated that at the concentration of 1.40 mg/mL of the pretreated, simultaneously treated, and posttreated proteins exhibited different antiviral efficacy of 56.50%, 65.72%, and 100% inhibition, respectively (Figure 4).

### 3.5. IC_50_ of the Posttreated Protein on Influenza A/New Caledonia/20/99 H1N1, A/Fujian/411/01 H3N2, and A/Thailand/1 (KAN-1)/2004 H5N1

**Figure 5:**
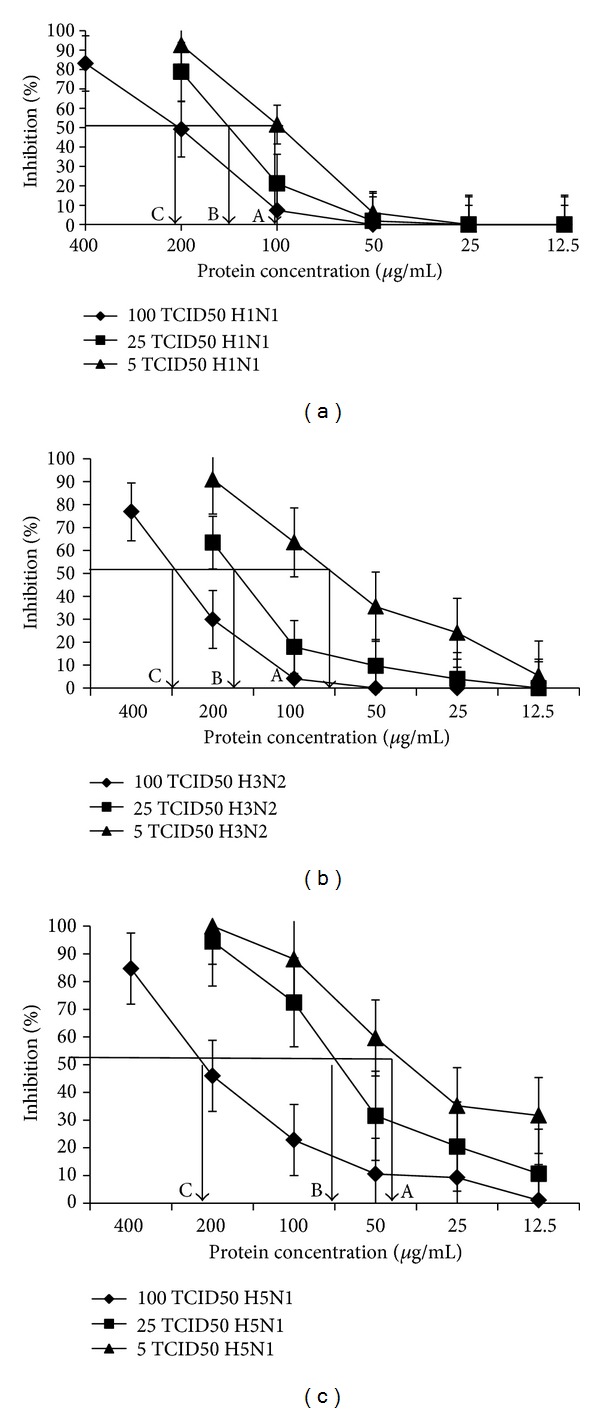
The IC_50_ of the posttreated protein on influenza A/New Caledonia/20/99 H1N1, A/Fujian/411/01 H3N2, and A/Thailand/1 (KAN-1)/2004 H5N1 ((a), (b), and (c), resp.). Using 5, 25, and 100 TCID_50_, the IC_50_ was calculated by graphically plotting the % inhibition of viral growth as a function of the protein concentrations.

The IC_50_ values of the posttreated protein against different subtypes of influenza A were determined. The results showed that at 5, 25, and 100 TCID_50_ of influenza A/New Caledonia/20/99 H1N1, A/Fujian/411/01 H3N2, and A/Thailand/1 (KAN-1)/2004 H5N1, the IC_50_ values were calculated to be 100, 150, and 200; 75, 175, and 300; and 40, 75, and 200 *μ*g/mL, respectively (Figure 5).

## 4. Discussion

*Momordica charantia* was reported to possess several antiviral activities including hepatitis B virus, dengue virus, and Human Immunodeficiency Virus. In our laboratory, we discovered that the protein purified from the ripe seeds of *M. charantia* contained effectively anti-HIV-1 activity [20]. For the present work, we have determined the novel antiviral property of the protein from this plant. At the concentration of 1.401 mg/mL, the protein did not exhibit any cytotoxicity in MDCK cells but effectively inhibited H1N1 as a dose-dependent manner (Figures 2 and 3). For the incubation time effect of the protein on H1N1, the posttreated protein displayed more potent antiviral action than the pretreated and simultaneously treated protein. This information implies that the protein of *M. charantia* can be effectively used after patients are being exposed to the virus. Interestingly, we found that the posttreated protein of 1.401 mg/mL completely inhibited at least 10^7^ FFU/mL of H1N1 (data not shown). This discovery confirmed the high antiflu activity of the protein of *M. charantia*.

To further investigate the antiviral activity of the plant protein, the MDCK cells were prior infected with Caledonia/20/99 H1N1, A/Fujian/411/01 H3N2, and A/Thailand/1 (KAN-1)/2004 H5N1 and later treated individually with the postprotein. As expected, the protein strongly inhibited not only H1N1 but also H3N2 and H5N1 subtypes. Its inhibitory effect was almost equally potent against the three viral subtypes. At the high dose of 100 TCID_50_, the protein still exhibited effective anti-influenza activity. Taken together with the former finding of its anti-HIV property, we believe that the protein of *M. charantia* possess the broad antiviral activity against various types of viruses in addition to HIV and influenza. According to previous publications, the antiviral and anticancer activities of the proteins belong to the action of the protein called ribosomal inactivating proteins (RIPs). So far, several RIPs have been identified from the plants in the Cucurbitaceae family such as MAP30 from *M. charantia* and GAP31 from *Gelonium multiflorum* [20, 23]. Additionally, our recent finding indicates that Cochinin B, a novel RIP purified from the seeds of *Momordica cochinchinensis*, contained a potent antitumor activity [24]. Nevertheless, whether the anti-influenza activity of the protein from *M. charantia* is associated with the action of RIP or not, further investigation is needed.

## 5. Conclusion

We have found that the protein purified from *M. charantia* possessed effective antiviral activity to a broad range of influenza A subtypes including H1N1, H3N2, and H5N1. Thus, this plant protein holds a great promise to be developed as an effective therapeutic agent against various and even new emerging subtypes of influenza A such as H7N9, which is now pandemic in China.
